# Runners Adapt Different Lower-Limb Movement Patterns With Respect to Different Speeds and Downhill Slopes

**DOI:** 10.3389/fspor.2021.682401

**Published:** 2021-06-29

**Authors:** David Sundström, Markus Kurz, Glenn Björklund

**Affiliations:** ^1^Sports Tech Research Centre, Department of Quality Management and Mechanical Engineering, Mid Sweden University, Östersund, Sweden; ^2^Swedish Winter Sport Research Centre, Department of Health Sciences, Mid Sweden University, Östersund, Sweden

**Keywords:** biomechanics, gait analysis, gradient, motion analysis, running technique, work economy

## Abstract

The aim of this study was to investigate the influence of slope and speed on lower-limb kinematics and energy cost of running. Six well-trained runners (VO_2max_ 72 ± 6 mL·kg^−1^·min^−1^) were recruited for the study and performed (1) VO_2max_ and energy cost tests and (2) an experimental running protocol at two speeds, 12 km·h^−1^ and a speed corresponding to 80% of VO_2max_ (V80, 15.8 ± 1.3 km·h^−1^) on three different slopes (0°, −5°, and −10°), totaling six 5-min workload conditions. The workload conditions were randomly ordered and performed continuously. The tests lasted 30 min in total. All testing was performed on a large treadmill (3 × 5 m) that offered control over both speed and slope. Three-dimensional kinematic data of the right lower limb were captured during the experimental running protocol using eight infrared cameras with a sampling frequency of 150 Hz. Running kinematics were calculated using a lower body model and inverse kinematics approach. The generic model contained three, one, and two degrees of freedom at the hip, knee, and ankle joints, respectively. Oxygen uptake was measured throughout the experimental protocol. Maximum hip extension and flexion during the stance phase increased due to higher speed (*p* < 0.01 and *p* < 0.01, respectively). Knee extension at the touchdown and maximal knee flexion in the stance phase both increased on steeper downhill slopes (both *p* < 0.05). Ground contact time (GCT) decreased as the speed increased (*p* < 0.01) but was unaffected by slope (*p* = 0.73). Runners modified their hip movement pattern in the sagittal plane in response to changes in speed, whereas they altered their knee movement pattern during the touchdown and stance phases in response to changes in slope. While energy cost of running was unaffected by speed alone (*p* = 0.379), a shift in energy cost was observed for different speeds as the downhill gradient increased (*p* < 0.001). Energy cost was lower at V80 than 12 km·h^−1^ on a −5° slope but worse on a −10° slope. This indicates that higher speeds are more efficient on moderate downhill slopes (−5°), while lower speeds are more efficient on steeper downhill slopes (−10°).

## Introduction

Running is one of the most popular physical activities, both recreationally and competitively. It is also a fundamental part of human locomotion and has been investigated in numerous studies (Cavanagh and Lafortune, 1980; Cavanagh and Kram, 1985; Staab et al., 1992; Anderson, 1996; Townshend et al., 2010; Kasmer et al., 2013). Competitive running includes many disciplines, categorized by distance or duration, in combination with various terrain and course surfaces. As in any locomotive endurance sport, long-distance running performance is determined by the athlete's maximum aerobic power (VO_2max_), lactate threshold, and work economy (Joyner, 1991; Joyner and Coyle, 2008). Several studies have investigated various biomechanical aspects of work economy in running (i.e., energy cost of running; Tartaruga et al., 2012; Santos-Concejero et al., 2014a, 2017).

Studies on the influence of foot-strike patterns on marathon performance show the dominance of the heel-strike pattern, irrespective of a runner's location on a course or the final race result (Kasmer et al., 2013). Although the heel-strike pattern is the dominant foot-strike pattern at all performance levels, there is a greater percentage of fore-foot runners among the fastest runners in level-terrain races (Hasegawa et al., 2007). 2D video recordings assessed this distribution of foot-strike patterns; however, no information of the inter- or intraindividual reliability is presented for the method itself. Furthermore, none of these studies include spatiotemporal stride characteristics nor angles of the lower extremities that could possibly explain differences in foot-strike patterns.

The benefit of an optimal foot-strike pattern is that it decreases the braking forces acting on the foot at the ground contact. These braking forces inherently counteract the propulsive forces that move the body in a forward running direction. Interestingly, the effect of foot-strike patterns on energy cost of running is not clear (Moore, 2016). One major reason for the uncertainty is the position of the lower limbs in relation to foot and possibly ankle flexibility. If the foot is placed too far in front of the hip, a runner is, by definition, over striding, which increases braking force (Lieberman et al., 2015). To decrease the risk of over striding, a runner should increase stride frequency (SF) with a concomitant decrease in stride length (SL). This may explain why an increase in SF decreases energy cost (Hunter and Smith, 2007; de Ruiter et al., 2014) at a given submaximal speed.

Overall performance on hilly and undulating trail-running courses is related to both uphill and downhill running ability. However, in trail running, which includes hilly terrain and sustained downhill sections with rocky and root-covered surfaces, downhill running ability seems to be more important to performance than uphill running ability (Kay, 2014). The results of Kay (2014) also showed that the fastest runners on these types of trail-running courses overall also excelled on the downhill sections. However, no analysis or explanation is presented as to why or how these runners achieved higher speeds of descent. Moreover, previous studies of downhill running show that oxygen uptake drops at a −5% gradient (approximately−3°) or steeper, despite speed increasing and runners performing at the maximal effort (Staab et al., 1992; Born et al., 2017). Therefore, it is reasonable to assume that biomechanics is a constraint on running speed and that this becomes more severe on steeper downhill slopes. Hence, lower-limb dynamics ought to be highly important to describe downhill running performance. Running on an instrumented treadmill at 3.0 m·s^−1^ shows that braking force peaks (parallel ground reaction forces) and braking impulses increase on steeper downhill slopes (Gottschall and Kram, 2005). Nevertheless, the study by Gottschall and Kram (2005) does not present any data to explain if and how running kinematics are altered to achieve higher braking forces on steeper downhill slopes. However, Buczek and Cavanagh (1990) showed that greater downhill slope was associated with greater knee flexion. Moreover, Khassetarash et al. (2020) showed that greater speed was associated with a greater hip angle range of motion, at both level and downhill slopes.

On level terrain, Lieberman et al. (2015) showed that the horizontal position difference between the ankle and hip at touchdown increases with greater speed. Nonetheless, neither Gottschall and Kram (2005) nor Lieberman et al. (2015) investigated near-race speed of high-performance athletes. Therefore, the aim of this study was to investigate the influence of downhill slope (0° to −10°) and speed (12 km·h^−1^ and speed at 80% of VO_2max_) on lower-limb kinematics and energy cost when running close to race pace. Hence, we hypothesized that (a) Increased running speed will have a negative effect on energy cost of running compared to slower running speed on the same slope; (b) increased downhill slope is associated with greater knee flexion; and (c) increased running speed is associated with greater range of motion in the hip joint.

## Methods

### Participants

Six well-trained male runners (VO_2max_: 72 ± 6 mL·kg^−1^·min^−1^, body mass: 71 ± 8 kg, body height: 178 ± 6 cm) were recruited for the study. They were all used to trail running in hilly terrain, including downhill, although their preferable running discipline varied between road running, trail running, and orienteering, including off-trail running. The participants were informed of the aim, procedures, and risks of the tests before giving their informed written consent to participate in the study. The regional ethical review board in Umeå, Sweden, preapproved the research techniques and experimental protocol (#2017/140-31), which conformed to the Declaration of Helsinki.

### General Design

The overall study comprised of two parts: the pretests and the experimental tests. The pretests included basic anthropometric measurements as well as submaximal and maximal treadmill-running protocols to determine participants' energy cost of running and VO_2max_. The experimental tests included a 30-min treadmill protocol to assess lower-limb kinematics and energy cost in level and downhill running.

### Pretests

Anthropometric measurements, including body height (cm) and body mass (kg), were conducted using a measuring tape and precision scale (SECA, Hamburg, Germany), respectively. The runners started the test with a 10-min warm-up on a motorized treadmill (Rodby Innovation AB, Vänge, Sweden) at a self-selected speed. Their energy cost (expressed as J·kg^−1^·m^−1^) was estimated using the Weir equation (Weir, 1949) and by measuring their steady-state oxygen uptake (VO_2_) during the final minute of a 5-min running period at 16 km·h^−1^. VO_2_ and respiratory exchange ratio (RER) were measured using the ergospirometry system Moxus Metabolic Cart (AEI Technologies, Pittsburg, PA, United States). To be accepted as a valid energy cost estimate, VO_2_ data had to meet the criterion of RER <1.00, indicating purely aerobic exercise. In addition to oxygen cost of running (VO_2_), we calculated the energy cost of running because it accounts for the different metabolic substrate mixtures when running at submaximal speeds and is more sensitive to changes in speed than oxygen cost (Fletcher et al., 2009). Maximal oxygen uptake (VO_2max_) was assessed during a ramp test, starting at 16 km·h^−1^ on a level running surface followed by a stepwise increase in slope of 1°/min until voluntary exhaustion. To confirm that the maximal effort was achieved, two criteria had to be satisfied: a rating of perceived exertion on the Borg scale >18 directly after completing the ramp test and RER >1.15. Runners breathed through a mouthpiece while wearing a nose clip to secure all expired air flowed through the ergospirometry system. The O_2_ and CO_2_ sensors were calibrated using two-component high-precision gases (%O_2_ = 15.99, %CO_2_ = 4.5 and %O_2_ = 21.00, %CO_2_ = 0.03, respectively), i.e., two-point calibration. The volume transducer was calibrated using a 3-L syringe (Hans Rudolph) for low, medium, and high flow rates. At all times during the use of the treadmill, a suspended safety harness connected to an emergency stop triggered by their bodyweight secured the participants.

### Experimental Tests

#### Test Protocol

The runners started the test with a 15-min warm-up at a self-selected speed on the same treadmill as used for the pretests. During the warm-up, the runners were familiarized with the three different slopes that would be used in the experimental testing (0°, −5°, and −10°). They then performed a treadmill running test at each of the three slopes, 0°, −5°, and −10°, at two different speeds, a baseline speed of 12 km·h^−1^ and 80% of the speed at which they achieved their VO_2max_ for level running (V80). V80 is close to race pace but still reliable to calculate the energy cost of running (RER <1.0) (Shaw et al., 2014). The participants ran each of the six slope–speed conditions for 5 min, a total running duration of 30 min. The experimental conditions were run in a randomized order to control any confounding factors, i.e., learning effect, and there were no pauses between the experimental conditions. Measurements of VO_2_ and energy cost of running were taken as previously described for the pretests.

#### Kinematics

Eight infrared cameras (Qualisys AB, Göteborg, Sweden, 300/301), evenly distributed around the treadmill (measurement volume: 2.0 × 2.0 × 2.0 m) and set to a capture frequency of 150 Hz, captured three-dimensional kinematic data of a full-body marker set consisting of 77 markers. For the present study, we only analyzed the pelvis and right lower limb (Karamanidis et al., 2003). Markers were attached to the following bony landmarks: both spina iliaca anterior superiors, both spina iliaca posterior superiors, lateral and medial femoral epicondyles, lateral and medial malleolus, most prominent point of the tuber calcanei, head of the first and fifth metatarsal bones, and top of the hallux. Furthermore, two rigid-plate marker clusters, each containing four markers, were mounted to the thigh and shank using hook-and-loop fasteners. For every speed–slope combination, we measured five trials. The measurements took place at the last 15 s of each minute during the 5-min efforts. On average, 89 ± 15 steps were then extracted and analyzed with respect to hip and knee angle in the sagittal plane for every participant and experimental condition.

### Data Analysis

Cardiorespiratory data were analyzed in Excel 2013 (Microsoft Office, v15.0). All data synchronization was performed in MATLAB R2016a (The MathWorks, Inc., Natick, MA, United States).

**Figure 1:**
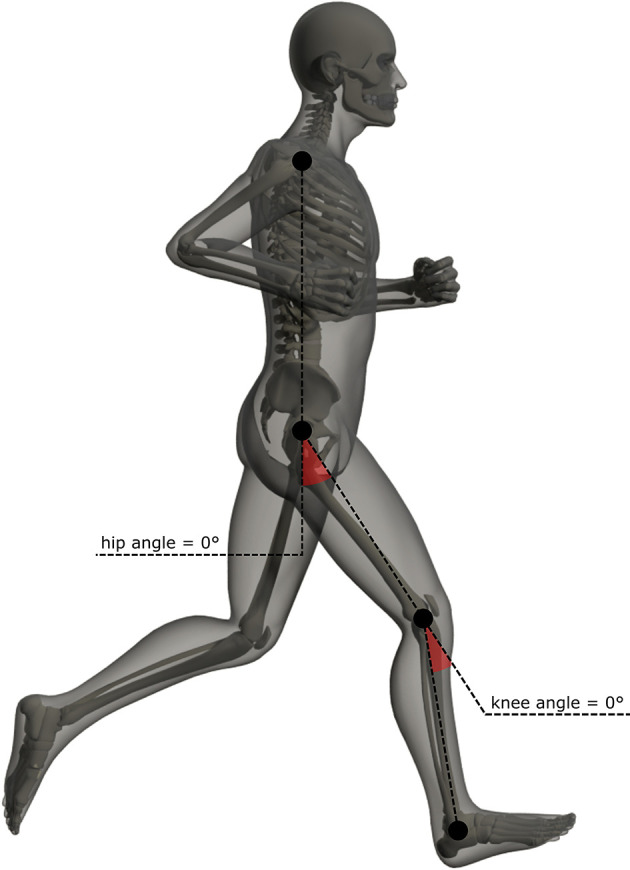
Lower-limb kinematic angles. Areas shaded red indicate the angles analyzed (sagittal plane of the hip and knee joint, respectively). Filled black circles closest to the red areas represent the joint center of the hip and knee. Dashed lines indicate 0° for the angles.

Hip and knee angles (Figure 1) were calculated using OpenSim 4.1 (Delp et al., 2007; Seth et al., 2018). We used a lower-body model (Gait2392_Simbody) with three, one, and two degrees of freedom in the hip, knee, and ankle joints, respectively. The generic models were scaled to each participant's mass and the position of the markers placed at their bony landmarks (scaling markers). OpenSim uses an inverse kinematic approach to calculate joint angles, and the kinematic data were filtered using a third-order zero-phase low-pass Butterworth filter with a cutoff frequency of 20 Hz.

We calculated gait events (i.e., touchdown and toe off) using kinematic algorithms (Fellin et al., 2010; Handsaker et al., 2016, respectively) and normalized the parameters with respect to the stance phase. Ground contact time (GCT) and SF were calculated using these gait events together with the treadmill speed, while SL was calculated according to Cavanagh and Williams (1982).

### Statistical Analyses

All data were checked for normal distribution using Shapiro–Wilk tests and assumption of homogeneity of variance *via* Levene's test. The data were processed and further analyzed using *jamovi* (version 1.2 [Computer Software]. Retrieved from https://www.jamovi.org) and MATLAB R2016a (The MathWorks, Inc., Natick, MA, United States). Two-way factorial ANOVA with repeated measures (speed × slope) was applied to test global differences for dependent variables as kinematic (hip-, knee-flexion extension), spatiotemporal (GCT, SL, SF), and cardiorespiratory (relative and absolute VO_2_, J·kg^−1^·m^−1^). For all ANOVAs, data were controlled for type one errors using Mauchly's sphericity test and, if violated, the Greenhouse–Geisser-corrected *F*-values were used. If there were global significances in the ANOVA, a further Bonferroni *post-hoc* analysis was performed. Generalized eta-squared ($\eta_{\text{G}}^{2}$) was used to determine the effect size for the ANOVA. The thresholds for interpreting the effect size were small: $\eta_{\text{G}}^{2}$ >0.02; medium: $\eta_{\text{G}}^{2}$ >0.13; and large: $\eta_{\text{G}}^{2}$ >0.26 (Bakeman, 2005). A paired Student's *t*-test was used to compare the differences in speed between 12 km·h^−1^ and V80 with Cohen's *d* as an effect size. Data are presented as mean ± SD or a 95% confidence interval (95% CI). The significance level was set to α = 0.05 a priori.

## Results

The speed at V80 of 15.8 ± 1.3 km·h^−1^ was considerably faster than the low-speed condition of 12 ± 0.0 km·h^−1^ (*p* < 0.001, 95% CI 2.45–5.24, *d* = 2.89).

### Spatiotemporal Parameters

Table 1 shows the changes in spatiotemporal parameters for the two different speeds and three different slopes. GCT was shorter at V80 compared with 12 km·h^−1^ throughout, independent of slope, as shown by the large effect size (Table 1). There was an interaction effect for speed and slope (*p* = 0.003; Table 1), while slope did not affect GCT (*p* = 0.714; Table 1). SL increased due to the faster running speed (*p* = 0.003) (large η G2: large) (Table 1) and further increased with a steeper slope, from −5° to −10° (*p* = 0.047), at V80 (η G2: medium) (Table 1). There was an overall decrease in SF for both running speeds when the steepness of the slope increased from 0° to −10° (*p* = 0.002) (η G2: medium) (Table 1).

**Table 1:** Spatiotemporal variables on level and downhill running (*n* = 6).

	**0^**°**^**	**−5^**°**^**	**−10^**°**^**	***F*-values, *P*-values, and effect size ($\eta_{\text{G}}^{2}$)**
**GCT (s)**				
12 km·h^−1^	0.262 ± 0.023	0.260 ± 0.032	0.259 ± 0.037	^a^*F*_(1,5)_ = 28.1, *p* = 0.003, $\eta_{\text{G}}^{2}$ = 0.312
80% of VO_2max_	0.221 ± 0.025^*^	0.222 ± 0.023^*^	0.230 ± 0.025^*^	^b^*F*_(2,10)_ = 0.3, *p* = 0.714, $\eta_{\text{G}}^{2}$ = 0.002
				^c^*F*_(2,10)_ = 5.3, *p* = 0.027, $\eta_{\text{G}}^{2}$ = 0.009
**SL (m)**				
12 km·h^−1^	2.50 ± 0.15	2.66 ± 0.21	2.66 ± 0.20^†^^#^	^a^*F*_(1,5)_ = 27.5, *p* = 0.003, $\eta_{\text{G}}^{2}$ = 0.689
80% of VO_2max_	3.30 ± 0.50^*^	3.33 ± 0.22^*^	3.68 ± 0.40^*^^†^^#^	^b^*F*_(2,10)_ = 9.3, *p* = 0.005, $\eta_{\text{G}}^{2}$ = 0.139
				^c^*F*_(2,10)_ = 1.3, *p* = 0.180, $\eta_{\text{G}}^{2}$ = 0.065
**SF (min**^**−1**^**)**				
12 km·h^−1^	80.3 ± 4.9	75.6 ± 5.8	75.6 ± 5.3^†^	^a^*F*_(1,5)_ = 0.1, *p* = 0.755, $\eta_{\text{G}}^{2}$ = 0.001
80% of VO_2max_	80.9 ± 7.1	79.5 ± 5.5	72.2 ± 6.0^†^	^b^*F*_(2,10)_ = 11.9, *p* = 0.002, $\eta_{\text{G}}^{2}$ = 0.210
				^c^*F*_(2,10)_ = 1.4, *p* = 0.282, $\eta_{\text{G}}^{2}$ = 0.075

*The values are presented as means ± SD. GCT, ground contact time; SL; stride length; SF; stride frequency*.

*A factorial ANOVA for repeated measurement was used to compare the speed and slope with a Bonferroni post-hoc test*.

a *Factorial ANOVA for repeated measurement of speed (2)*.

b *Factorial ANOVA for repeated measurement of slope (3)*.

C *Interaction effect between speed and slope (2 × 3)*.

* *Statistically different from 12 km·h^−1^*.

† *Statistically different from 0°*.

# *Statistically different from −5°*.

### Hip and Knee Angles

Table 2 displays the hip and knee angles for the speed and slope conditions studied. During stance, maximal hip flexion and extension increased at V80 compared to 12 km·h^−1^ regardless of the slope (*p* = 0.007) (both η G2: medium) (Table 2). Maximal knee flexion during stance was greater at a −10° slope compared with both 0° (*p* = 0.004) and −5° (*p* = 0.008) (both η G2: medium) (Table 2). There was a small but non-significant interaction effect for speed and slope on knee flexion at touchdown (*p* = 0.077) (η G2: small) (Table 2). Knee flexion at touchdown decreased with increases in the steepness of the decline slope (*p* < 0.001) (η G2: large) (Table 2).

**Table 2:** Hip and knee angles on level and downhill running (*n* = 6).

	**0^**°**^**	**−5^**°**^**	**−10^**°**^**	***F*-values, *P*-values, and effect size ($\eta_{\text{G}}^{2}$)**
**Hip max (°)**				
12 km·h^−1^	24.2 ± 3.2	24.7 ± 4.9	22.0 ± 3.7	^a^*F*_(1,5)_ = 19.5, *p* = 0.007, $\eta_{\text{G}}^{2}$ = 0.168
80% of VO_2max_	26.7 ± 4.2^*^	27.1 ± 3.5^*^	27.2 ± 4.5^*^	^b^*F*_(2,10)_ = 1.3, *p* = 0.309, $\eta_{\text{G}}^{2}$ = 0.021
				^c^*F*_(2,10)_ = 1.5, *p* = 0.261, $\eta_{\text{G}}^{2}$ = 0.029
**Hip min (°)**				
12 km·h^−1^	−18.9 ± 3.9	−19.6 ± 4.7	−19.6 ± 5.2	^a^*F*_(1,5)_ = 11.6, *p* = 0.019, $\eta_{\text{G}}^{2}$ = 0.147
80% of VO_2max_	−23.1 ± 2.9^*^	−22.0 ± 4.3^*^	−23.4 ± 5.9^*^	^b^*F*_(2,10)_ = 0.2, *p* = 0.821, $\eta_{\text{G}}^{2}$ = 0.005
				^c^*F*_(2,10)_ = 1.5, *p* = 0.269, $\eta_{\text{G}}^{2}$ = 0.008
**Knee max (°)**				
12 km·h^−1^	50.3 ± 3.9	50.9 ± 4.5	53.5 ± 3.6^†^^#^	^a^*F*_(1,5)_ = 2.3, *p* = 0.193, $\eta_{\text{G}}^{2}$ = 0.007
80% of VO_2max_	51.5 ± 4.0	51.5 ± 4.4	53.5 ± 3.3^†^^#^	^b^*F*_(2,10)_ = 12.0, *p* = 0.002, $\eta_{\text{G}}^{2}$ = 0.090
				^c^*F*_(2,10)_ = 2.8, *p* = 0.111, $\eta_{\text{G}}^{2}$ = 0.005
**Knee TD (°)**				
12 km·h^−1^	20.6 ± 3.8	16.7 ± 4.2^†^	15.4 ± 5.7^†^	^a^*F*_(1,5)_ = 0.3, *p* = 0.632, $\eta_{\text{G}}^{2}$ = 0.003
80% of VO_2max_	21.6 ± 4.2	16.5 ± 3.4^†^	13.4 ± 4.2^†^	^b^*F*_(2,10)_ = 34.0, *p* < 0.001, $\eta_{\text{G}}^{2}$ = 0.334
				^c^*F*_(2,10)_ = 3.4, *p* = 0.077, $\eta_{\text{G}}^{2}$ = 0.026

*The values are presented as means ± SD. Hip max, maximum hip flexion; HIP min, maximum hip extension; Knee max, maximum knee flexion; Knee TD, knee flexion at touchdown*.

*A factorial ANOVA for repeated measurement was used to compare the speed and slope with a Bonferroni post-hoc test*.

a *Factorial ANOVA for repeated measurement of speed (2)*.

b *Factorial ANOVA for repeated measurement of slope (3)*.

c *Interaction effect between speed and slope (2 × 3)*.

* *Statistically different from 12 km·h^−1^*.

† *Statistically different from 0°*.

# *Statistically different from −5°*.

### Cardiorespiratory Parameters

**Figure 2:**
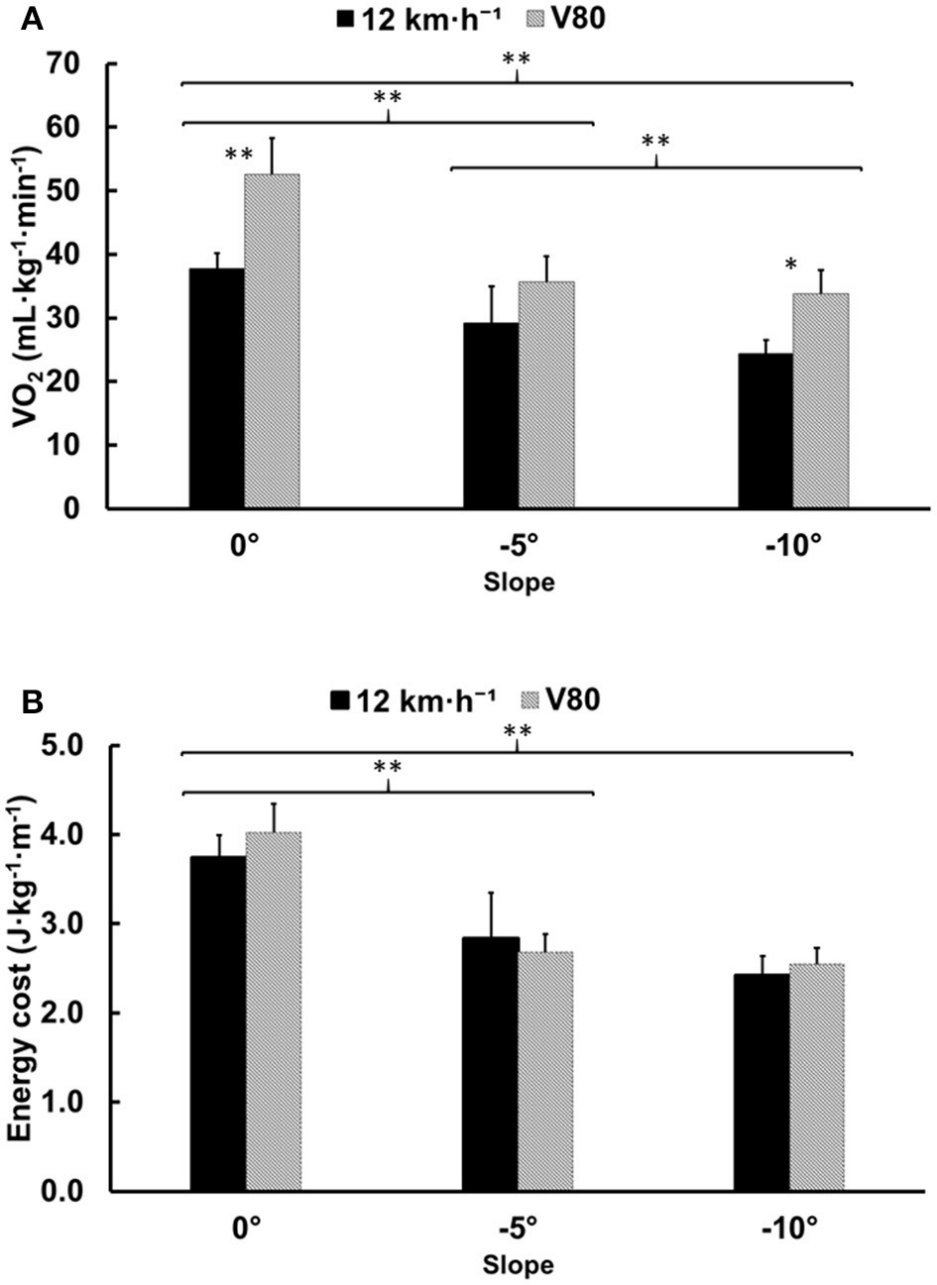
**(A)** Relative oxygen uptake (mL·kg^−1^·min^−1^) for 0°, −5°, and −10° slopes. **(B)** Energy cost of running for 0°, −5°, and −10° slopes expressed as joules relative to body mass per meter (J·kg^−1^·m^−1^). Running speeds of 12 km·h^−1^ and 80% of VO_2max_ are illustrated using black and gray bars, respectively. Brackets illustrate the differences between slopes, while * or ** above the bars indicate differences between speeds (*p < 0.05 and **p < 0.01). The values given are mean ± SD.

Relative VO_2_ (mL·kg^−1^·min^−1^) increased at faster speeds [*F*_(1,5)_ = 27.8, *p* = 0.003, $\eta_{\text{G}}^{2}$ = 0.637: large] but decreased during running on steeper downhill slopes [*F*_(2,10)_ = 87.9, *p* < 0.001, $\eta_{\text{G}}^{2}$ = 0.761: large; Figure 2A]. There was a medium interaction effect for speed and slope [*F*_(2,10)_ = 7.9, *p* = 0.009, $\eta_{\text{G}}^{2}$ = 0.167: medium] that is explained by the higher relative VO_2_ at V80 compared with 12 km·h^−1^ at slopes of 0° and −10°, but not at −5° (Figure 2A). Absolute VO_2_ (L·min^−1^) was greater at V80 than 12 km·h^−1^ [*F*_(1,5)_ = 24.4, *p* = 0.004, $\eta_{\text{G}}^{2}$ = 0.367: large] but decreased with a change in slope of 0° to −5° [*F*_(2,10_ = 61.6, *p* < 0.001, $\eta_{\text{G}}^{2}$ = 0.506: large]. However, absolute VO_2_ did not decrease further between −5° and −10° slopes (*p* = 0.123). There was a small interaction effect for speed and slope that is explained by the higher absolute VO_2_ at V80 compared with 12 km·h^−1^ on a slope of 0° and −10° but not −5° [*F*_(2,10)_ = 8.8, *p* = 0.006, $\eta_{\text{G}}^{2}$ = 0.063: small]. Energy cost of running (J·kg^−1^·m^−1^) remained unchanged between the two speeds [*F*_(1,5)_ = 0.9, *p* = 0.379, $\eta_{\text{G}}^{2}$ = 0.023: small] but improved with a steeper downhill slope [*F*_(2,10)_ = 90.1, *p* < 0.001, $\eta_{\text{G}}^{2}$ = 0.826: large; Figure 2B]. Energy cost of running showed no interaction effect for speed × slope [*F*_(2,10)_ = 3.5, *p* = 0.071, $\eta_{\text{G}}^{2}$ = 0.096: small; Figure 2B].

## Discussion

The main finding of the study is a shift in the energy cost of running, not only due to changes in downhill slope but speed as well. This shift seems to imply that energy savings at steep declines as compared to level running are greater at 12 km·h^−1^ than at a speed equivalent to 80% of the runner's VO_2max_ (V80). This supports hypothesis (a).

Minetti et al. (2002) derived a fifth-order polynomial relationship between energy cost of running and gradient, showing that the downhill gradient of minimal energy cost is close to −20% (equal to a slope angle of −11.3°). However, the energy cost of running in that study was measured at slower speeds than are typical in races (~10–11 km·h^−1^ in the gradient range 0 to −20%) and the polynomial relationship does not account for differences in speed. Moreover, their study investigates neither the effect of speed nor the interaction effect of gradient and speed on energy cost. Although we did not find any significant interaction effect of slope and speed on energy cost of running, the results do show an interaction effect for slope and speed on relative VO_2_. On a moderate downhill slope of −5°, the energy cost of running was higher at 12 km·h^−1^ than at V80. On the steeper downhill slope of −10°, the relationship was reversed, and the energy cost of running was higher at V80 compared with 12 km·h^−1^. Although this was not significant, it indicates that speed, not just slope, may alter the energy cost of downhill running, especially on steep slopes. The current experimental evidence suggest that minimal energy cost occurs with flatter slopes as individuals run at faster speeds.

Gottschall and Kram (2005) found that the parallel breaking force impulse increases linearly with steeper decline gradients, while the parallel propulsive force impulse decreased exponentially at an ever-decreasing rate in relation to the downhill gradient. According to Gottschall and Kram (2005), this may be why running becomes more metabolically costly beyond a −20% gradient. In accordance with this, Vernillo et al. (2020) found higher propulsive force impulse and higher step frequency at 4.17 m·s^−1^ than at 2.50 and 3.33 m·s^−1^ on a −10° slope. Another possible explanation is less pronounced elastic energy storage and release in downhill running compared with level (Snyder et al., 2012). At faster speeds, due to insufficient muscle contraction velocity, the parallel braking impulse may increase and thus require an increased parallel propulsive force impulse. Moreover, the greater propulsive force demanded requires muscle contractions of greater force. These high-force contractions recruit additional fast-twitch muscle fibers that are less energy efficient (Coyle et al., 1992). Both these mechanisms may explain the shift in downhill slope of minimal energy cost toward less-steep slopes. Furthermore, higher energy cost of running at high speed on steep descents may be explained by the greater range of motion in the knee observed in the present study, since this is known to be associated with higher knee power absorption during the stance phase (DeVita et al., 2008).

In accordance with Park et al. (2019), the present study showed that knee flexion increased with steeper downhill slope, and therefore, we accept hypothesis (b). Seki et al. (2020) showed similar results between level and downhill running for maximal hip extension (level: 167° ± 13 vs. downhill: 168° ± 13). Additionally, in accordance with Khassetarash et al. (2020), the present study displayed that hip angle range of motion increased with running speed, and therefore, hypothesis (c) is accepted. The increased hip angle range of motion at higher speeds is also associated with an increase in SL. SL at V80 further increased at a slope between −5° and −10°, which could, partly, be explained by the corresponding increase in knee angle extension at touchdown (20.6° ± 3.8 vs. 16.7° ± 4.2 vs. 15.4° ± 5.7). This running technique adaption, often called over striding, may also be responsible for the greater energy cost of running at high speeds on steep descents mentioned above. Over striding at high speeds on steep descents may also be a strategy to reduce work demand on the hip flexor and extensor muscles while managing speed and avoiding uncontrolled acceleration. Supporting this hypothesis, DeVita et al. (2008) found that the lever arm of the ground reaction force is greater in uphill running than in downhill, suggesting that downhill running does not exert more strain on the hip muscles than uphill running, despite the lower magnitude of ground reaction force in uphill running. This might be why Park et al. (2019) did not find increased joint power in the hip joint on downhill slopes.

The increased range of motion of the knee in steep downhill compared with level running, as observed in the present study, could be explained by the increased knee extension at touchdown. Increased range of motion in the knee and greater knee extension at touchdown (Buczek and Cavanagh, 1990: 24.6 ± 3 vs. 17.0 ± 4.2) on steeper downhill slopes are both consistent with previous findings on downhill running (Buczek and Cavanagh, 1990; Mizrahi et al., 2001). Furthermore, Vernillo et al. (2020) found a slope × speed interaction effect for peak ground reaction forces in the normal direction with the highest values at 4.17 m·s^−1^ and −10°, together with an increase in breaking impulse for the same slope–speed combination, which could be explained by over striding. However, we cannot analyze nor confirm those kinetic findings and the effect of over striding, because we did not measure ground reaction forces, in the present study.

Pacing strategy is an important consideration, especially in long-distance races. Given the tendency for higher energy costs in high-speed steep downhill running (V80, −10°) compared with slower speeds (12 km·h^−1^, −10°), a wise strategy may be to reduce speed on steep downhill slopes to retain metabolic energy. This is in contrast with the common regime of pacing strategy in endurance sports that favors an even work rate and therefore high-speed descents and slow-speed ascents. Another argument in support of the slow-speed steep downhill running strategy is the increased muscle damage caused by prolonged high-impact eccentric exercise, such as downhill running (Sargeant and Dolan, 1987). In the present study, GCT decreased at faster running speeds consistently across all slopes, but we found no effect of slope on GCT. Moreover, GCT at 12 km·h^−1^ was in line with previously reported GCT in outdoor downhill running (Björklund et al., 2019). In the literature, there are equivocal findings presented regarding the relationship between GCT and running economy (i.e., energy cost or oxygen cost of running). Several studies found no association between GCT and running economy (Heise and Martin, 2001; Kyröläinen et al., 2001; Støren et al., 2011), while some found that longer GCT correlates with better running economy (Di Michele and Merni, 2014), and others found that shorter GCT correlates with better running economy (Nummela et al., 2007; Santos-Concejero et al., 2014b).

The speed at 80% of VO_2max_ (V80) of 15.8 ± 1.3 km·h^−1^ is close to the most commonly used reference speed of 16 km·h^−1^ when assessing oxygen cost of running (Barnes and Kilding, 2015). The mean value of VO_2_ at V80 in level running of 52.5 ml·kg^−1^·min^−1^ is in line with the values reported for highly trained male runners (mean: 50.6, range: 40.5–66.8 ml·kg^−1^·min^−1^) (Conley and Krahenbuhl, 1980; Daniels and Daniels, 1992; Morgan et al., 1994; Saunders et al., 2004). Moreover, the mean value of VO_2_ at 12 km·h^−1^ in level running of 37.7 ml·kg^−1^·min^−1^ is in line with the values reported for moderately trained male runners (mean: 40.7, range: 37.4–48.1 mL·kg^−1^·min^−1^) (Johnson et al., 1997; Spurrs et al., 2003; Støren et al., 2008; Berryman et al., 2010; Mikkola et al., 2011).

Study limitations include the low number of participants (*n* = 6). Despite the low number of participants, the effects for the measured variables were estimated to be reasonably large according to the sample size calculation using a power of 0.8 with an alpha at 0.05. Nevertheless, the generalization of the study results should be related to runners that are used to run on trails and undulating terrain. The surface itself do pose a constraint on the applicability of the current study results in trail running. Therefore, future studies may investigate the validity of these indoor treadmill-running findings for in-field trail running on ever-changing surfaces. Furthermore, the steepest slope (−10°) might have not been steep enough to see the full effect of how speed influences the running economy at various slopes. However, according to previous studies using slower speeds, the slope used in the current study was estimated to be a turning point where the energy cost of running is minimal. The additional measurements of ground reaction forces in future studies could provide more insight into how joint moments are changing with respect to different speeds and slopes in treadmill running.

## Conclusion

The results of this study show that runners modify their hip movement pattern in the sagittal plane during the stance phase with changes in speed, whereas they alter their knee movement pattern during the touchdown and stance phases with respect to the slope. Therefore, runners competing in hilly races may benefit from training programs that include running on race-specific slopes at race speed. We also observed that running economy was better at moderate speeds than near-race speed on steep downhill slopes (−10°), while the reverse was true on less-steep declines (−5°). This implies that pacing schemes for different race distances have to be taken into consideration during preparation, e.g., low-speed steep descents to retain metabolic energy in long-distance races.

## Data Availability Statement

The raw data supporting the conclusions of this article will be made available by the authors, without undue reservation.

## Ethics Statement

The studies involving human participants were reviewed and approved by the regional ethical review board in Umeå, Sweden. The patients/participants provided their written informed consent to participate in this study.

## Author Contributions

DS and GB designed the study and performed the experiments. DS, GB, and MK performed the data analysis, interpreted the results, revised the manuscript, approved the final version, and agreed to be accountable for all aspects of the study. DS wrote the first draft. All authors contributed to the article and approved the submitted version.

## Conflict of Interest

The authors declare that the research was conducted in the absence of any commercial or financial relationships that could be construed as a potential conflict of interest.
